# Impact of Organic Solvents, Surfactants, and Nonfunctionalized PEGs on PEGylation Reaction Yield

**DOI:** 10.1021/acsomega.6c06664

**Published:** 2026-09-22

**Authors:** Raissa H. S. Florindo, Andrea M. Salgado, Vivian M. Saez, Tito L. Moitinho-Alves, Jose A. Ramon

**Affiliations:** † PEQ/COPPE, Universidade Federal do Rio de Janeiro, Cidade Universitária, Rio de Janeiro, Rio de Janeiro 21941-909, Brazil; ‡ Department of Biochemical Engineering, School of Chemistry, Universidade Federal do Rio de Janeiro, Rio de Janeiro, Rio de Janeiro 21941-909, Brazil; § Department of Analytical Chemistry, Chemical Institute, 28125 Universidade Federal do Rio de Janeiro, Rio de Janeiro, Rio de Janeiro 21941-909, Brazil

## Abstract

The optimization of PEGylation reaction yield is critical for the efficient production of therapeutic bioconjugates. This study systematically investigates the influence of three distinct classes of additives -organic cosolvents, nonreactive poly­(ethylene glycols) (PEGs) of varying molecular weights, and surfactants- on the conjugation efficiency of a model protein. Quantitative analysis via SDS-PAGE densitometry revealed that the addition of 40% (v/v) DMSO significantly enhanced the reaction yield compared to the aqueous control, while methanol resulted in a marked decrease in efficiency. These results were rationalized using Hansen solubility parameters, which indicated that the high dispersion cohesion (18.4 MPa^1/2^) of DMSO promotes superior solvation of PEG chains, thereby minimizing competitive hydrolysis pathways. Furthermore, the inclusion of nonfunctionalized PEGs (4 and 8 kDa) provided a marginal increase in conjugation, likely due to the excluded volume effect. In contrast, the anionic surfactant SDS severely inhibited the reaction, a phenomenon attributed to electrostatic repulsion between the charged SDS-PEG-protein complexes. The nonionic surfactant polysorbate 20 exhibited a neutral effect on yield, suggesting its utility primarily as a protein stabilizer without compromising conjugation. These findings provide a comprehensive framework for the strategic selection of additives to modulate the physicochemical environment and maximize PEGylation efficiency.

## Graphical abstract

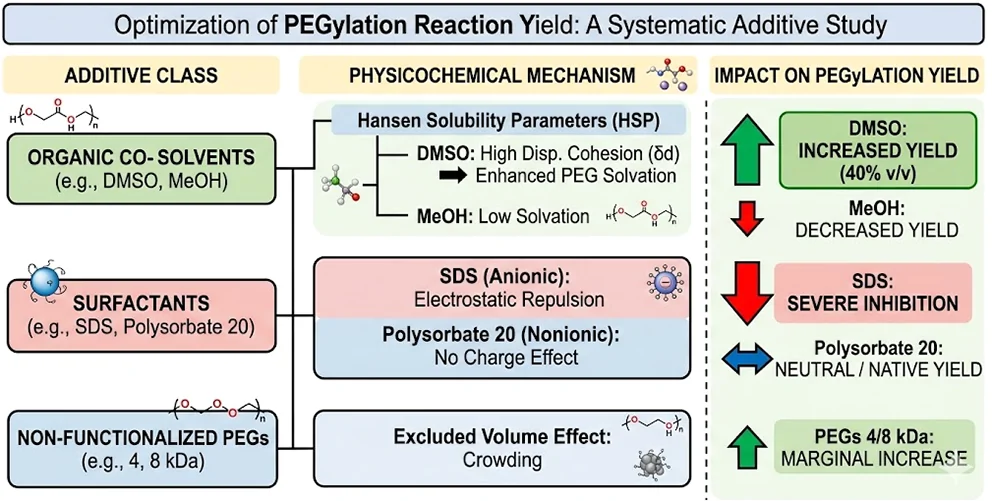

## 1 Introduction

Therapeutic proteins have been utilized for decades to treat a wide array of pathologies, a trend that has accelerated significantly following advancements in recombinant protein technology.^1^ However, the clinical translation of these proteins is often hindered by several inherent challenges, ranging from suboptimal pharmacokinetics and susceptibility to proteolytic degradation to the activation of the host immune system.^2–4^ To mitigate these limitations, various strategies have been developed, including controlled-release systems based on biodegradable microparticles and conjugation to biocompatible polymers. Among these approaches, the covalent conjugation of poly­(ethylene glycol) (PEGylation) has emerged as the most successful strategy, yielding approximately 30–35 approved products worldwide-depending on regional regulatory approvals and classification criteria (i.e., distinct brand names versus active conjugated entities).^5,6^ In contrast, particulate carriers have demonstrated limited clinical translation for macromolecular therapeutics; polymer-based microparticles account for only a single historically approved protein formulation, while liposomal platforms remain largely restricted to the delivery of small molecules and nucleic acids.^7,8^

However, the PEGylation reaction is complex and involves PEG properties, reaction conditions and conjugation strategies.^9–11^ Unlike approaches found in the literature on the subject, this research focuses on how the composition of the reaction medium affects reaction efficiency. Thus, the effects of organic solvents, surfactants and nonreactive polymers on the yield of the random PEGylation of recombinant l-asparaginase using succinimidyl carbonate-activated mPEG (12 kDa).

Protein PEGylation has been a mainstay in biopharmaceutical development since the 1970s.^12^ This process involves the conjugation of poly­(ethylene glycol) (PEG) to diverse biomolecules, including proteins, peptides, enzymes, antibody fragments, oligonucleotides, and synthetic drugs.^13^ The primary benefits of PEGylation include enhanced physicochemical properties, such as reduced protein aggregation and increased solubility, as well as an extended *in vivo* circulatory half-life and protection against proteolytic enzymes. These advantages are primarily attributed to the significant increase in the hydrodynamic volume of the PEG-conjugated biomolecules.^14,15^ However, comparing published studies on the PEGylation reaction is complicated by the lack of standardized end points for evaluating performance. Depending on the objectives of each study, authors may report protein conversion,^16^ mono-PEGylation yield,^17,18^ recovery after purification,^19–21^ or residual biological activity,^17,22^ making direct comparisons difficult.

Despite its widespread use, the conjugation of a protein to PEG is a highly complex process. On one hand, parallel reactions occur between various susceptible conjugation sites and the involved solvents. On the other hand, successive reactions can lead to the formation of multi-PEGylated conjugates. Consequently, a broad range of factors influence the yield of the desired product. For instance, the excluded volume effect (EVE) and the hydrolysis of active PEG in aqueous solutions are critical factors that can compromise reaction efficiency. The excluded volume generated by PEG may induce the formation of protein-rich and PEG-rich nanodomains, thereby reducing the frequency of productive collisions between proteins molecules and activated PEG. Consequently, the excluded volume effect is mechanistically relevant because it alters the local distribution and effective concentration of macromolecules in solution, influencing both protein solubility and PEGylation efficiency.^23^ Functionalized PEG reagents bearing NHS ester or succinimidyl carbonate moieties, which are broadly applied in protein PEGylation, are subject to rapid hydrolytic degradation at basic pH. Specifically, under typical conditions such as pH 9.0, the reported half-life of PEG-NHS is on the order of 9 min.^11^ To compensate for these losses, a significant molar excess of mPEG is often required, which increases production costs and complicates downstream purification processes.^23^ While numerous studies have sought to optimize PEGylation by adjusting parameters such as PEG properties, reaction conditions (pH, temperature, reaction time, and PEG-protein molar ratio), and conjugation strategies,^24–26^ relatively little attention has been paid to the specific composition of the reaction medium—particularly the role of organic solvents, surfactants, and nonreactive polymers.

These three classes of reaction-medium components were selected because they can affect PEGylation through distinct physicochemical mechanisms. Organic solvents may modify the polarity of the reaction medium and affect protein solvation and conformational stability;^27–30^ surfactants may affect protein stability, aggregation and the accessibility of reactive groups;^31–33^ and nonreactive PEGs may modify macromolecular interactions through excluded volume and crowding effects.^23^ Other reaction parameters, such as pH, temperature, PEG-to-protein molar ratio, ionic strength and reaction time are also important determinants of PEGylation efficiency but were outside the specific scope of this study.

Previous investigations into reaction medium engineering for PEGylation have largely centered on organic cosolvents to modulate protein conformation and reaction selectivity. For instance, the incorporation of DMSO, ethanol, or methanol in microbial transglutaminase-mediated PEGylation of salmon calcitonin and human growth hormone shifted product distribution toward mono-PEGylated species without compromising bioactivity.^34^ Similarly, the addition of 2-butanol in the PEGylation of interferon β-1b increased overall and mono-PEGylation yields by over 37% and 36%, respectively, compared to purely aqueous media.^35^

Beyond organic solvents, however, systematic investigations into other medium additives remain notably scarce. Surfactants have been extensively evaluated for their postconjugation impact on protein stability, conformation, and aggregation suppression,^36,37^ yet their direct role in modulating PEGylation reaction kinetics and yields remains largely unaddressed. Likewise, the deliberate use of nonreactive crowding agents or macromolecular depletants to steer conjugation efficiency has been barely explored; an isolated exception is the work by Meziadi et al.,^23^ who utilized nonreactive 0.35 kDa mPEG to exploit excluded volume effects and promote coupling between proteins and 12 kDa PEG-SC.

However, these previous studies primarily investigated individual components of the reaction medium or specific aspects of protein behavior. To the best of our knowledge, no systematic comparison of the effects of organic solvents, surfactants and nonreactive PEGs on PEGylation efficiency in a common reaction system has been reported.

The composition of the reaction medium can profoundly influence protein structure, reagent accessibility, and reaction kinetics. Organic solvents may alter the conformation and solubility of proteins, while surfactants can modulate surface exposure and aggregation behavior.^38–41^ Furthermore, the presence of nonreactive PEG may influence the reaction efficiency through the excluded volume effect or by altering macromolecular interactions.^23^

Despite these potential impacts, comprehensive studies on how these components influence the efficiency of PEG conjugation remain limited. Therefore, this study aimed to investigate the influence of reaction medium composition on the yield of the PEGylation reaction. Specifically, we evaluated the effects of various solvents, surfactants, and nonreactive PEG to determine their impact on process efficiency and optimize the production of protein conjugates.

## 2 Materials and Methods

### 2.1 Materials

Luria–Bertani (LB) broth and succinimidyl carbonate-activated mPEG (12 kDa) were purchased from Sigma-Aldrich (St. Louis, MO, USA). Kanamycin was obtained from Gibco (Grand Island, NY, USA). All other chemical reagents and analytical-grade solvents were sourced from Merck (Darmstadt, Germany).

### 2.2 Production and Purification of l-Asparaginase

l-asparaginase (ASNase) was produced in-house following the protocol previously described by Ramon et al.^42^ Briefly, recombinant *Escherichia coli* BL21 (DE3), harboring the *Zymomona mobilis* l-asparaginase gene under the regulation of the *lac* operon promoter, was cultured in 2 L of LB broth. Upon reaching the desired density, cells were harvested and subsequently lysed using a high-pressure homogenizer (APLAB-10–1.000 bar, Artepeças, Taubaté, Brazil). The crude extract was purified via successive chromatographic stages, specifically immobilized metal affinity chromatography (IMAC; HisTrap HP, 5 mL) followed by size-exclusion chromatography (SEC; HiTrap Desalting, 5 mL).

### 2.3 PEGylation Assays

#### 2.3.1 Effect of Organic Cosolvents

The influence of organic cosolvents on the PEGylation reaction was evaluated in 1.5 mL microtubes. The reaction mixture consisted of 50 μL of the respective organic solvent, 50 μL of ASNase solution (1.1 mg/mL), and 25 μL of functionalized PEG (4 mg/mL in ice-cold 1 mM HCl). The medium was maintained under constant agitation for 2 h.

#### 2.3.2 Effect of Nonfunctionalized PEG and Surfactants

To assess the influence of nonfunctionalized PEG and surfactants, the procedure described above was modified. Instead of organic solvents, 50 μL of either nonactivated PEG (5%) or surfactant (2.4%) was added to the mixture. Both additives were prepared in 66 mM phosphate buffer at pH 8.0.

### 2.4 Electrophoretic Analysis (SDS-PAGE)

Protein profiles and PEGylation yields were analyzed via Slab-SDS-PAGE using 12% linear acrylamide gels. Following electrophoresis, proteins were visualized using Coomassie Brilliant Blue staining. Quantitative densitometric analysis of the stained gels was performed using ImageJ software (National Institutes of Health, USA).

### 2.5 Statistical Analysis

The influence of experimental variables on PEGylation yield was assessed by analysis of variance (ANOVA), which enables the partitioning of total variability into within-group and between-group components to identify statistically significant differences among group means. When significant effects were detected (*p* < 0.05), Fisher’s Least Significant Difference (LSD) post hoc test was employed to perform pairwise comparisons between treatments. All statistical analyses were conducted using Statgraphics Centurion XVII (StatPoint Inc., USA).

## 3 Results and Discussion

### 3.1 Influence of Organic Cosolvents on PEGylation Efficiency

The PEGylation reaction was investigated in the presence of five organic cosolvents: acetonitrile, DMSO, ethanol, isopropanol, and methanol. These solvents were selected based on their established use in the literature for modulating protein–polymer conjugation.^34,43^ In addition, some of these solvents have already been used in PEGylation reactions. Peng et al.,^35^ for example, used DMSO in the PEGylation of granulocyte colony-stimulating factor with SC-PEG, and the reaction yield increased by 42% compared to the reaction in an aqueous solution. DMSO, ethanol, and methanol have also been tested in the PEGylation of proteins with monomethoxy poly­(ethylene glycol) amine, using the enzyme transglutaminase for site-specific PEGylation. In the presence of these solvents, only mono-PEGylated conjugates were produced, whereas in buffer solution a mixture of mono- and di-PEGylated conjugates were obtained.^34^

For the statistical analysis, the fraction of residual native protein was selected as the primary response variable to provide a standardized, robust metric of overall substrate conversion across all experimental runs. The percentage of unreacted native protein was quantified by SDS-PAGE densitometry using ImageJ and evaluated in Statgraphics to assess the main effects of the reaction medium components. In parallel, mono-, di-, and tri-PEGylated species were individually quantified to assess product distribution, conjugate heterogeneity, and reaction selectivity under each condition.

The electrophoretic profile of the conjugation products in the presence of different organic cosolvents is presented in Figure 1, with the corresponding densitometric quantification of native and PEGylated species summarized in Table 1. Visual inspection of the SDS-PAGE gel demonstrates the appearance of distinct conjugated bands corresponding to mono- (1-PEG, ∼55–65 kDa), di- (2-PEG, ∼85–100 kDa), and tri-PEGylated forms (3-PEG, >120 kDa), alongside the unreacted native ASNase monomer (∼35 kDa).

**Figure 1:**
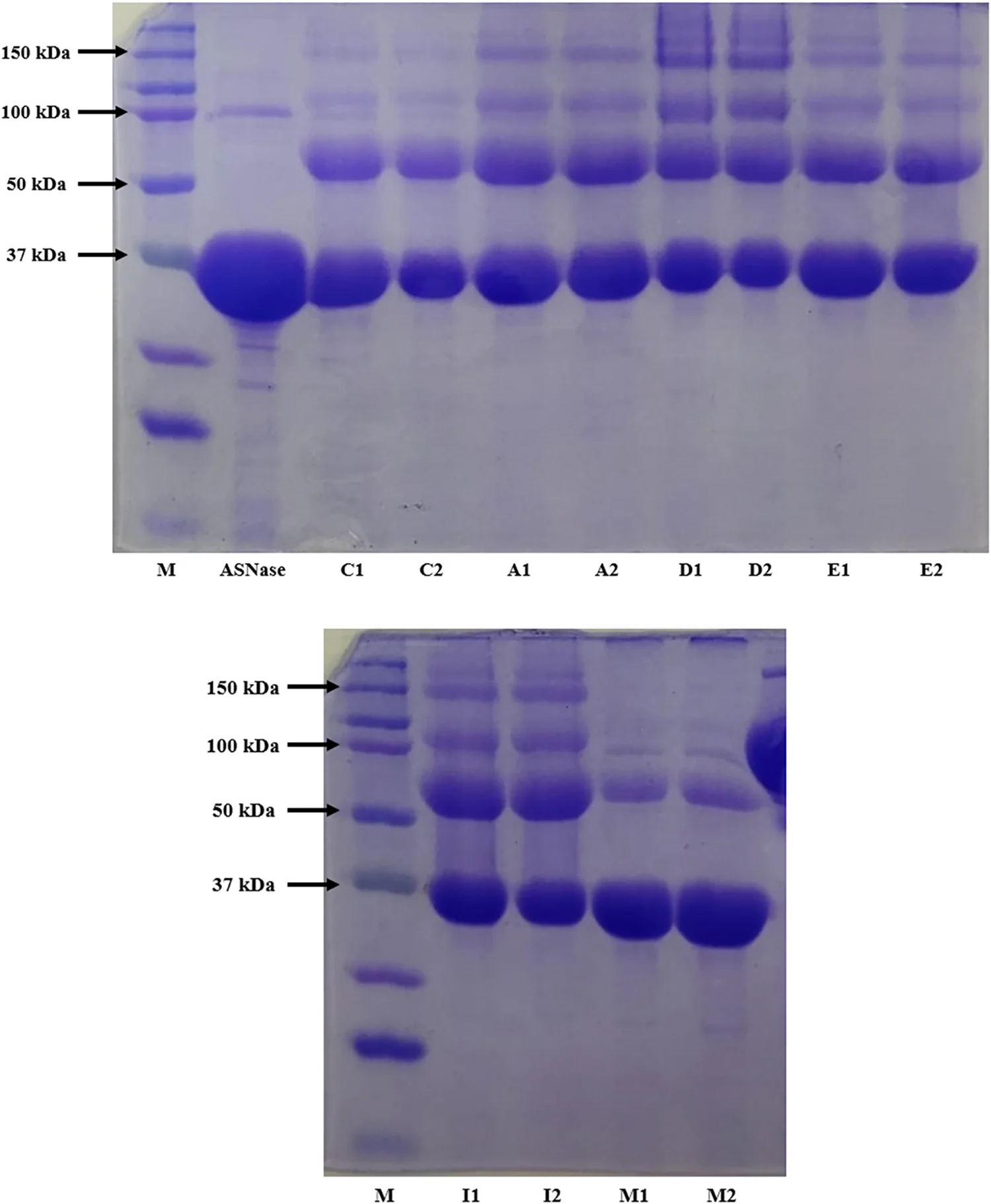
12% polyacrylamide gel showing the products of the conjugation reaction in different solvents. (M) molecular weight marker; (C) control; (A) acetonitrile; (D) DMSO; (E) ethanol; (I) isopropanol; and (M) methanol. Lanes 1 and 2 represent duplicate samples.

**Table 1:** Effect of Organic Cosolvents on the Fraction of Residual Native Protein (%), Determined by Densitometric Analysis of Electrophoresis Gels^a^ ^,^ ^b^

	control	acetonitrile	DMSO	ethanol	isopropanol	methanol
Native	63 ± 5	61 ± 2	49 ± 7	60 ± 5	57 ± 2	86 ± 4
1-PEG	34 ± 5	34 ± 3	33 ± 5	34 ± 4	36 ± 4	14 ± 4
2-PEG	3.0 ± 0.9	3.1 ± 0.8	9.9 ± 0.9	2.9 ± 0.2	4 ± 2	n.d.
3-PEG	1.3 ± 0.6	3 ± 1	8 ± 2	4 ± 1	3 ± 1	n.d.

a n.d.: not detected.

b Values are presented as mean ± standard deviation (SD) from four determinations (*n* = 4), obtained from two independent experimental days with duplicate measurements performed on each day.

The overall conjugation efficiency, evaluated in terms of substrate conversion (lower residual native protein), followed the descending order: DMSO > isopropanol > ethanol ≈ acetonitrile ≈ buffer control > methanol (Table 1). Notably, the addition of DMSO yielded the highest extent of bioconjugation, reducing the residual native protein fraction to 49 ± 7%. In contrast to other cosolvents where mono-PEGylation was the predominant modification (∼34–36%) with negligible higher conjugates (<4%), DMSO promoted multi-PEGylation, leading to significantly higher yields of 2-PEG (9.9 ± 0.9%) and 3-PEG (8 ± 2%) species, which is clearly visualized by the intense multiband pattern in lanes D1–D2 (Figure 1).

Isopropanol, ethanol, and acetonitrile displayed comparable conversion profiles, yielding 57 ± 2%, 60 ± 5%, and 61 ± 2% residual native protein, respectively, which was similar to the aqueous control reaction (63 ± 5%) (lanes C, A, E, and I in Figure 1). Conversely, methanol exerted a pronounced detrimental effect on conjugation, resulting in the highest fraction of unreacted protein (86 ± 4%), with only 14 ± 4% mono-PEGylated conjugate and undetectable levels of higher substituted species (lanes M1–M2).

One-way ANOVA confirmed statistically significant differences among the experimental groups (*p* < 0.0001). Subsequent posthoc analysis using Fisher’s LSD test (Table 2) revealed three distinct homogeneous groups: DMSO produced a statistically superior conjugation efficiency (group a) compared to all other conditions; isopropanol, ethanol, acetonitrile, and the buffer control formed an intermediate homogeneous subset with no mutually significant differences (group b); and methanol exhibited a significantly lower conjugation performance (group c).

**Table 2:** Statistical Comparison of the Residual Native Protein Fraction with Different Cosolvents

groups	residual native protein fraction (%)^a^
DMSO	49^a^ ± 7
Isopropanol	57^b^ ± 2
Ethanol	60^b^ ± 5
Acetonitrile	61^b^ ± 2
Control	63^b^ ± 5
Methanol	86^c^ ± 4

a Values are expressed as mean ± SD. Means sharing the same letter are not significantly different according to Fisher’s LSD test (*p* > 0.05).

#### 3.1.1 Mechanistic Considerations: Nucleophilicity vs Solubility

The conjugation of proteins with succinimidyl carbonate-functionalized poly­(ethylene glycol) (SC-PEG) involves a nucleophilic attack on the carbonate carbon by protein-based electron-donating groups, such as lysine residues. In purely aqueous systems, water serves as a competing nucleophile, leading to PEG hydrolysis. Consequently, replacing 40% of the aqueous buffer with an organic cosolvent was hypothesized to favor protein conjugation by suppressing this competitive hydrolysis pathway.

The nucleophilicity of the reaction medium could potentially influence PEGylation efficiency by promoting competing reactions with the activated PEG, thereby reducing the fraction of mPEG-SC available for reaction with protein nucleophiles. To assess whether this property could explain the differences observed among the solvents, reported Mayr nucleophilicity parameters (N) were considered. The reported values are 11.30 for DMSO (O-attack), 7.54 for methanol, 7.44 for ethanol, 6.49 for isopropanol and 2.23 for acetonitrile, corresponding approximately to the order DMSO > methanol ≈ ethanol > isopropanol > acetonitrile.^44–48^ However, this order did not correspond to the experimental PEGylation behavior observed in the present study. For example, DMSO, despite having the highest reported N value among the solvents evaluated, resulted in the lowest residual native protein, whereas methanol resulted in the highest residual native protein. These observations indicate that solvent nucleophilicity alone cannot account for the differences in PEGylation efficiency. Other solvent-dependent properties, such as PEG solubility, protein–solvent interactions, conformational effects and the stability of the activated PEG, may therefore contribute to the observed reaction behavior. It should be noted that Mayr N parameters are empirical and system dependent. Therefore, they are used here as a comparative descriptor of solvent nucleophilicity rather than as a direct quantitative predictor of reactivity toward mPEG-SC.

Instead, the effect likely relates to PEG solubility and conformation. PEGylation can be modeled as a series of successive pseudo-first-order reactions where the conformation of the macromolecules dictates the reaction rate. We employed the Hansen Solubility Parameter (HSP) method to evaluate these interactions.^49^ The total solubility parameter (δ_t_) is calculated as

$$\delta_{t}^{2}=\delta_{d}^{2}+\delta_{p}^{2}+\delta_{h}^{2}$$

In this equation, δ_d_, δ_p_, and δ_h_ correspond to the dispersive, polar, and hydrogen-bonding contributions to the cohesive energy, respectively. The Hansen Solubility Parameter (HSP) methodology utilizes the three cohesion parameters as spatial coordinates within a three-dimensional Hansen space. In this geometric model, a solvent is represented as a discrete point (δ_d,S_, δ_p,S_, δ_h,S_), while the polymer (solute) is depicted as a sphere or spheroid. The center of the polymer sphere corresponds to its characteristic coordinates (δ_d,P_, δ_p,P_, δ_h,P_), and its boundary is defined by the interaction radius (*jR*), which is numerically equivalent to the polymer’s total solubility parameter (δ_t,P_).

The fundamental solubility criterion dictates that for dissolution to occur, the coordinates of the solvent must reside within the volume of the polymer’s solubility sphere. Operationally, this is determined by calculating the distance (*ijR*) between the solvent point (*i*) and the center of the polymer sphere (*j*) using the following equation

Rij=[4(δd,S−δd,P)2+(δp,S−δp,P)2+(δh,S−δh,P)2]1/2

In this model, solubility is predicted when the distance is less than the interaction radius (*^ij^R* < *^j^R*). Conversely, if *^ij^R* > *^j^R*, the polymer is deemed insoluble in that specific medium.

An alternative and widely utilized expression of this criterion is the relative energy difference (RED), defined as

RED=RijRj

According to this normalized model, a RED < 1.0 indicates that the liquid acts as a favorable solvent for the polymer, whereas a RED > 1.0 characterizes “progressively poorer solvents”. The value RED = 1.0 represents the critical boundary condition for the system.

The Hansen solubility parameters (δ_d_, δ_p_ and δ_h_) of the solvents were obtained from tabulated literature values.^49^ For PEG, the parameters were obtained from the literature study by Özdemir and Güner,^50^ who calculated the Hansen parameters for PEG using different approaches. In the present study, the values obtained using the van Krevelen-Hoftyzer method (δ_d_ = 17.0, δ_p_ = 10.7 and δ_h_ = 8.9 MPa^1/2^) were used. Because these parameters were determined for PEGs with molecular weights up to 10 kDa, these values were used as literature-based estimates for the 12 kDa mPEG-SC employed in the present study. The results indicate that the solubility of PEG follows a decreasing trend: DMSO > acetonitrile ≈ isopropanol > ethanol > methanol ≫ water.

To explore the potential capacity of RED parameter to predict the reaction yield by means of the fraction of residual native protein a correlation analysis was done. The relationship between the calculated RED parameter and residual native protein (values reported in Table 1) was further evaluated across the five solvents investigated. A strong positive linear association was observed between RED and residual native protein (Pearson’s *r* = 0.912, *R* ^2^ = 0.831, *p* = 0.031), indicating that higher RED values tended to result in higher levels of residual native protein and, consequently, lower PEGylation conversion. Notably, the two solvents with the same RED value (RED = 0.4) exhibited relatively similar residual native protein (57 ± 2% and 61 ± 2%), supporting the relevance of RED parameter as a descriptor of solvent characteristics associated with reaction performance. Nevertheless, the relationship should be interpreted with caution because only five solvents were evaluated. Thus, the observed correlation provides quantitative support for the proposed relationship between RED and residual native protein, but additional solvents covering a broader range of RED values would be required to establish a general predictive relationship (Table 3).

**Table 3:** Analysis of PEG Solubility in the Reaction Medium, with and without Organic Cosolvents, Based on Hansen Parameters

solvent	δ_d_ ^a^ ^,^ ^b^	δ_p_ ^a^ ^,^ ^b^	δ_h_ ^a^ ^,^ ^b^	δ_T_ ^a^	*^ij^R* ^c^	RED^c^
Acetonitrile	15.3	18	6.1	24.4	8.7	0.4
DMSO	18.4	16.4	10.2	26.7	7.2	0.3
Ethanol	15.8	8.8	19.4	26.5	11.1	0.5
Isopropanol	15.8	6.1	16.4	23.6	9.0	0.4
Methanol	15.1	12.3	22.3	29.6	14.3	0.6
Water	15.5	16	42.3	47.8	34.3	1.6

a MPa^1/2^

b Source: Solvent parameters obtained from Hansen solubility parameters: a user’s handbook.^49^

c The *^ij^R* values were calculated using literature-reported Hansen solubility parameters for PEG from Özdemir and Güner, estimated using the van Krevelen-Hoftyzer method.^50^

#### 3.1.2 Thermodynamic Interpretation of Solvent-PEG Interactions

The analysis of poly­(ethylene glycol) (PEG) solubility through the lens of Hansen Solubility Parameters (HSP) provides a robust rationale for the observed variations in PEGylation yields across different solvent systems. This framework specifically elucidates why dimethyl sulfoxide (DMSO) facilitates the highest reaction yields and why the yields obtained with acetonitrile are statistically equivalent to those of isopropanol. The distinct contributions of dispersion, polar, and hydrogen-bonding forces within each solvent dictate the thermodynamic favorability of their interaction with the polymer.

##### 3.1.2.1 Role of Dispersion Forces (δ_d_)

The dispersion cohesion parameter (δ_d_) which arises from nonpolar London dispersion forces, is primarily influenced by molecular polarizability.^51^ Among the solvents investigated, DMSO exhibits the highest δ_d_ (18.4 MPa^1/2^), which serves as its dominant cohesive component. A characteristic consistent with its high polarizability and boiling point (189 °C).^52^ This substantial dispersion capacity enables DMSO to effectively solvate the apolar segments of the PEG chain, thereby facilitating the solubility of high-molecular-weight species such as the 12 kDa SC-PEG utilized in this study. In contrast, methanol possesses the lowest δ_d_ (15.1 MPa^1/2^) due to its small molecular size and limited polarizability. Consequently, its ability to interact with the apolar regions of the PEG backbone is significantly diminished, necessitating a greater reliance on other intermolecular forces.

##### 3.1.2.2 Role of Polar Interactions (δ_p_)

The polar cohesion parameter (δ_p_) stems from permanent dipole–dipole interactions (Hansen, 2007). Acetonitrile represents a unique case in this regard, as polar forces constitute its primary cohesive energy component due to the strong dipole moment of the cyano (C≡N) group.^53^ While PEG itself is polar and exhibits good solubility in acetonitrile, the solvent’s moderate dispersion forces and limited hydrogen-bond-accepting capacity may restrict its efficacy in solvating extended PEG chains. Conversely, the alcohols (methanol, ethanol, and isopropanol) demonstrate their lowest cohesive energy values in the polar parameter, as the hydroxyl dipole is partially offset by the nonpolar alkyl chain.^54^ DMSO maintains an intermediate δ_p_, attributed to the significant dipole of the S=O bond.^55,56^

##### 3.1.2.3 Role of Hydrogen-Bonding (δ_h_)

The hydrogen-bonding parameter (δ_h_) quantifies the capacity for H-bond interactions. Alcohols generally exhibit high δ_h_ values, with methanol displaying the greatest capacity in the current study.^49^ Functioning as both an H-bond donor and acceptor, methanol interacts strongly with the ether oxygens of the PEG backbone. This interaction typically favors the solubility of lower-molecular-weight PEGs, where hydrogen bonding is the predominant stabilizing force. DMSO, however, possesses the lowest δ_h_ value among the solvents. As a polar aprotic solvent, it can only accept hydrogen bonds via its oxygen atom and lacks donor capability. This restriction limits its H-bonding interactions with the polymer, further reinforcing its reliance on dispersion forces for effective solvation.

##### 3.1.2.4 The Aqueous Anomaly

A notable discrepancy in this analysis involves water, which served as experimental control. Although included in the comparative analysis, its calculated Hansen parameters yield implausible predictions, suggesting that PEG is insoluble in water—a conclusion that contradicts empirical evidence. This anomaly is attributed to the highly complex and structured nature of aqueous polymer solvation, where approximately two to three water molecules associate with each ethylene oxide repeating unit via specific hydrogen-bonding networks.^57–60^

Such molecular-level interactions are not explicitly captured by HSP framework, which provides a simplified representation of solvent–solute interactions and do not explicitly account for the dynamic and highly structured nature of the hydrogen-bonding network of water. This aspect may be particularly important in polymer-containing systems, in which hydration, association and molecular conformational effects also contribute to the observed behavior. For example, studies of PEG-water systems have shown that the Flory–Huggins interaction parameter depends on polymer concentration and temperature, with this behavior being associated, at least in part, with specific water-PEG interactions.^61–63^ Alternative approaches, such as modified Flory–Huggins models, polymer hydration models and association theories such as the Statistical Associating Fluid Theory (SAFT), may therefore provide a more detailed description of these interactions.^61,64^

### 3.2 Influence of Nonfunctionalized PEG Molecular Weight

The impact of nonreactive polyethylene glycol (PEG) on conjugation efficiency was evaluated using polymers of 3, 4, 8, and 20 kDa. Following the methodology established for the solvent effect analysis, the degree of conjugation was quantified via densitometric analysis of Coomassie Brilliant Blue–stained SDS-PAGE gels (Figure 2). The fraction of residual native protein served as the primary metric for evaluating the influence of these additives, where a higher native protein percentage indicates a reduction in conjugation yield. The experimental results are summarized in Figure 2 and Table 4.

**Figure 2:**
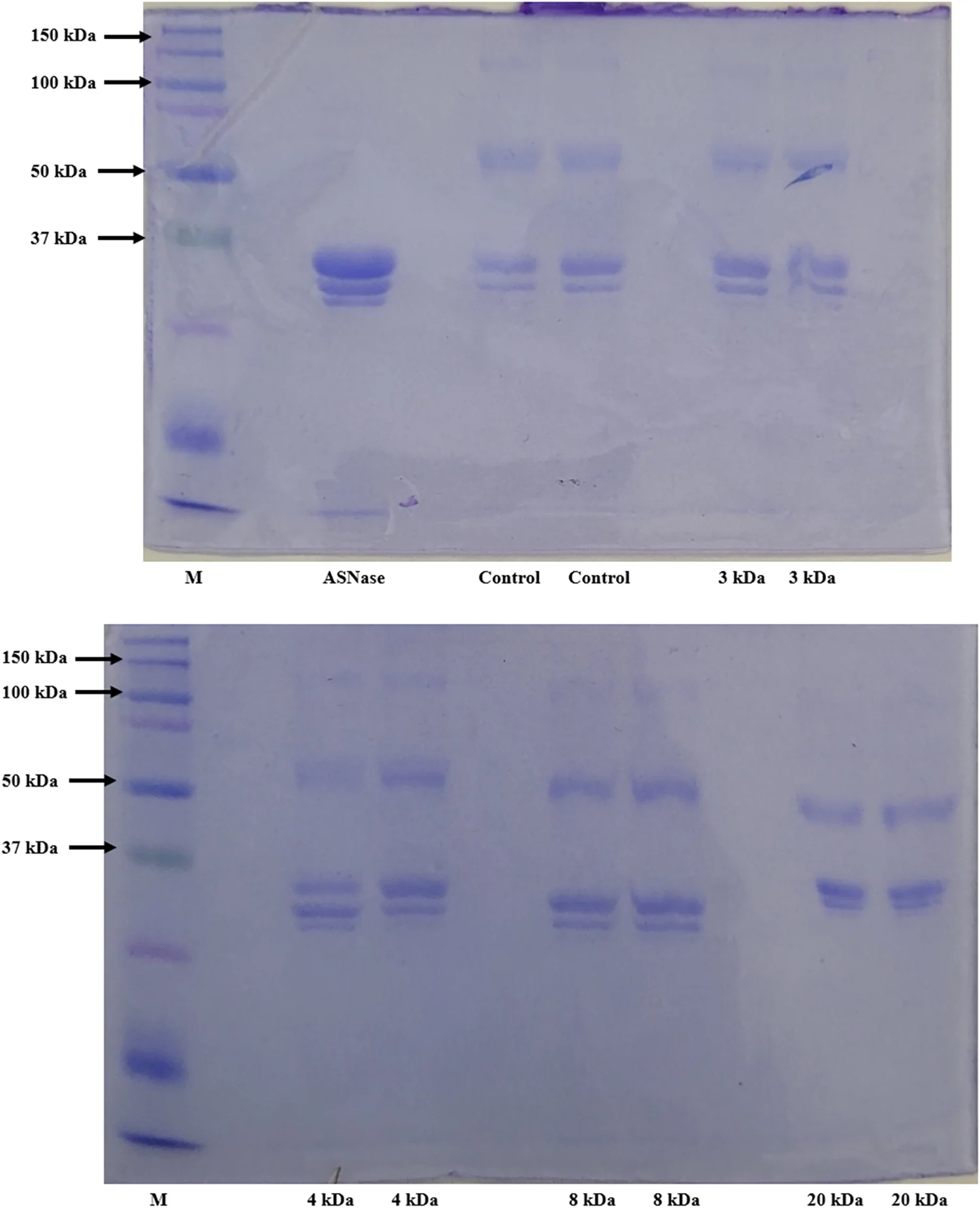
15% polyacrylamide gel showing the products of the conjugation reaction with nonreactive mPEG of different molecular weights: 3, 4, 8, and 20 kDa.

**Table 4:** Effect of Non-Reactive Poly­(ethylene glycol) on the Fraction of Residual Native Protein (%), Determined by Densitometric Analysis of Electrophoresis Gels^a^ ^,^ ^b^

	control	3 kDa	4 kDa	8 kDa	20 kDa
Native	51 ± 4	68 ± 3	51 ± 6	59 ± 11	69 ± 11
1-PEG	44 ± 2	30 ± 3	43 ± 9	31 ± 6	30 ± 11
2-PEG	5 ± 3	1 ± 1	4 ± 3	7 ± 3	n.d.
3-PEG	n.d.	1 ± 1	2 ± 2	4 ± 4	n.d.

a n.d.: not detected.

b Values are presented as mean ± standard deviation (SD) from four determinations (*n* = 4), obtained from two independent experimental days with duplicate measurements performed on each day.

One-way ANOVA indicated differences among the experimental groups (*p* < 0.03). According to Fisher’s LSD posthoc analysis (Table 5), the conjugation yield observed in the presence of 4 kDa nonreactive PEG showed no statistically significant deviations from the buffer control (51 ± 4 native protein). However, the inclusion of 3 kDa and 20 kDa nonfunctionalized polymers resulted in a higher native protein fraction (68% and 69%, respectively), indicating a measurable inhibitory effect on the total protein conversion. Finally, nonreactive PEG 8 kDa displayed an intermediate behavior relative to the other groups.

**Table 5:** Statistical Comparison of the Residual Native Protein Fraction with Different Non-Reactive PEGs

groups	residual native protein fraction (%)^a^
Control	51^a^ ± 4
4 kDa	51^a^ ± 6
8 kDa	59^ab^ ± 11
3 kDa	68^b^ ± 3
20 kDa	69^b^ ± 11

a Values are expressed as mean ± SD. Means sharing the same letter are not significantly different according to Fisher’s LSD test (*p* > 0.05).

Notably, a shift in the distribution of conjugates was observed in the 3–8 kDa range. While the buffer control reaction produced only mono- and di-PEGylated species, the addition of nonreactive PEGs within this molecular weight range facilitated the formation of higher-order conjugates (3-PEG), albeit in low proportions (1–4%).

This phenomenon can be partly explained by the excluded volume effect associated with macromolecular crowding, as previously characterized by Meziadi et al.^23^ However, kinetic effects cannot be excluded, as the presence of PEG may increase medium viscosity and consequently affect molecular diffusion. Since these effects were not independently evaluated in the present study, their relative contributions cannot be distinguished. While Meziadi et al.^23^ observed a generalized increase in PEGylation using extremely low molecular weight mPEG (0.35 kDa), the results in this study suggest that with larger nonfunctionalized species (3–8 kDa), this enhanced proximity primarily favors the deeper PEGylation of already modified protein molecules rather than increasing the initial conversion of the native enzyme.

Finally, the marked inhibitory effect observed with the 20 kDa nonfunctionalized PEG (69 ± 11% native protein) may be associated with changes in the physicochemical properties of the reaction medium. Higher molecular weight PEGs can increase the viscosity of aqueous solution,^65^ which may in turn affect molecular diffusion. Indeed, previous studies have reported an inverse relationship between protein translational diffusion and solution viscosity in polymer-containing media.^66^ Studies of PEG-crowded protein systems have also shown reduced protein diffusivity with increasing PEG concentration.^67^ However, viscosity was not directly measured in the present study, and diffusion coefficients and mixing behavior were not experimentally evaluated. Therefore, the lower conjugation efficiency observed with 20 kDa PEG should not be attributed solely to increased viscosity; diffusion and mass-transfer limitations may also contribute.

Overall, the addition of nonreactive PEGs did not lead to a consistent improvement in conjugation efficiency under the evaluated conditions. While PEG 4 kDa (51 ± 6%) showed no significant difference compared to the control (51 ± 4%), PEGs of 3 (68 ± 3%) and 20 kDa (69 ± 11%) appeared to negatively affect the reaction, and PEG 8 kDa (59 ± 11%) exhibited intermediate behavior. These results suggest that the effect of nonreactive PEG is dependent on its molecular weight and may not necessarily enhance PEGylation performance. Therefore, further studies are required to better understand the underlying mechanisms and to determine whether specific conditions or PEG characteristics could lead to beneficial effects.

**Figure 3:**
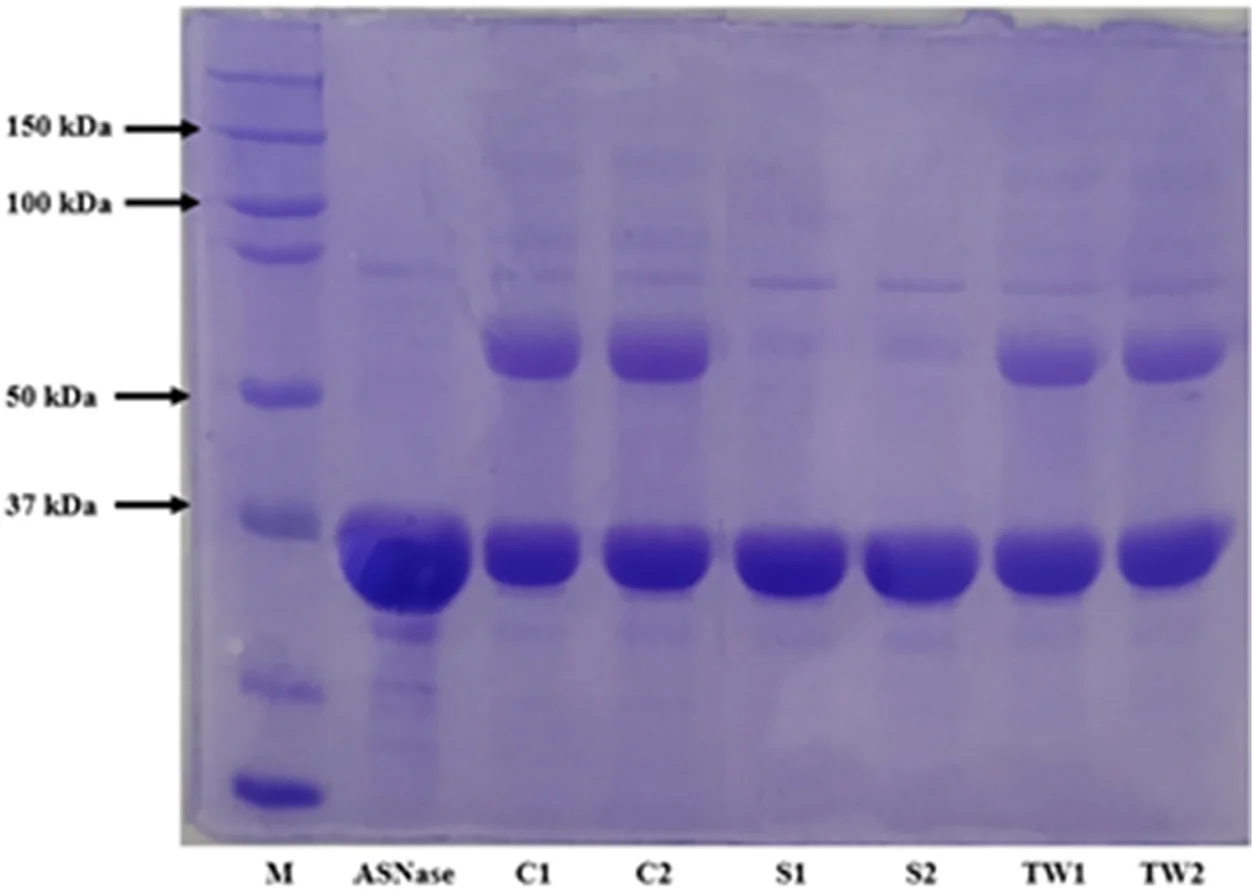
12% polyacrylamide gel showing the products of the first conjugation reaction in different surfactants. (M) molecular weight marker; (C) control; (P) PEG; (S) sodium dodecyl sulfate; (TW) polysorbate 20. Lanes 1 and 2 represent duplicate samples.

**Table 6:** Effect of Surfactants on the Fraction of Residual Native Protein (%), Determined by Densitometric Analysis of Electrophoresis Gels^a^

	buffer	SDS	polysorbate 20
Native	72 ± 5	97 ± 1	77 ± 4
1-PEG	25 ± 4	3 ± 1	22 ± 4
2-PEG	1.7 ± 0,6	n.d.	0.7 ± 0.3
3-PEG	0.8 ± 0.5	n.d.	0.9 ± 0.7

a Values are presented as mean ± standard deviation (SD) from four determinations (*n* = 4), obtained from two independent experimental days with duplicate measurements performed on each day.

### 3.3 Impact of Surfactants on Conjugation Efficiency

Surfactants are widely used in protein systems due to their ability to modulate intermolecular interactions and improve stability.^68–70^ However, their influence on PEGylation reactions remains poorly understood, particularly due to the limited number of studies addressing surfactant–protein–PEG interactions.^36^ Sodium dodecyl sulfate (SDS) and polysorbate 20 were selected based on their contrasting ionic profiles and their established utility in biochemical and pharmaceutical formulations. As with the previous evaluations of solvents and polymers, the degree of conjugation was quantified via densitometry of Coomassie Brilliant Blue–stained SDS-PAGE gels (Figure 3). The experimental findings are summarized in Table 6.

Statistical analysis (ANOVA) revealed significant differences among the experimental conditions (*p* = 0.0001). Posthoc pairwise comparisons using Fisher’s LSD test (Table 7) indicated that the presence of SDS (97 ± 1%) resulted in a significantly lower conjugation efficiency compared to all other groups. In contrast, the reaction yield in the presence of polysorbate 20 (77 ± 4%) closely resembled that of the buffer control (72 ± 5%), indicating that this nonionic surfactant does not substantially interfere with the conjugation process.

**Table 7:** Statistical Comparison of the Residual Native Protein Fraction with Different Surfactants

groups	residual native protein fraction (%)^a^
Control	72^a^ ± 5
Polysorbate 20	77^a^ ± 4
SDS	97^b^ ± 1

a Values are expressed as mean ± SD. Means sharing the same letter are not significantly different according to Fisher’s LSD test (*p* > 0.05).

#### 3.3.1 Mechanistic Analysis of Surfactant-Induced Effects

The literature regarding surfactant impact on bioconjugation remains limited; however, several mechanistic interpretations can be drawn from these results. The near-total inhibition of the conjugation reaction in the presence of SDS (97% residual native protein) is likely attributable to the specific interactions established between the anionic surfactant and the reaction components. SDS is known to form complexes with both the PEG polymer and the protein substrate through a combination of hydrophobic and electrostatic interactions. The formation of these polyanionic complexes imparts a significant negative charge to both the protein and the polymer. Consequently, strong electrostatic repulsion between the species hinders their proximity and prevents the nucleophilic attack required for covalent bond formation.^50,71–73^ While specialized biophysical assays (e.g., zeta potential measurements and CD spectroscopy) will be valuable to characterize the fine conformational and interfacial transitions in dedicated follow-up studies, the experimental reaction profiles reported here consistently corroborate this macromolecular repulsion model.

Conversely, polysorbate 20, a widely utilized nonionic surfactant, appears to facilitate a more favorable reaction environment. By improving solute dispersion in aqueous systems, polysorbates can reduce protein–protein interactions and mitigate aggregation phenomena.^74,75^ In addition, polysorbate 20 is known to stabilize proteins through preferential adsorption onto hydrophobic regions on the protein surface and by competing for interfacial sites, thereby reducing interfacial stress and aggregation.^31^ Although specific studies on l-asparaginase are limited, such mechanisms suggest a potential stabilizing effect on this enzyme. In contrast to ionic surfactants such as SDS, which strongly interact with proteins and may induce structural disruption, nonionic surfactants generally preserve native conformation. Thus, polysorbate 20 may contribute to maintaining protein structural integrity and accessibility of conjugation sites, balancing protein stability and reaction conditions.

The potential influence of the critical micelle concentration (CMC) must also be considered when interpreting the behavior of polysorbate 20. The final surfactant concentration used in this study (1.0% w/v) was substantially above its reported aqueous CMC (∼0.05–0.06 mM, corresponding to ∼0.006–0.007% w/v),^76^ confirming that polysorbate 20 was predominantly present as supramolecular micellar assemblies. Notably, statistical comparison revealed that polysorbate 20 yielded a residual native protein fraction (77 ± 4%) comparable to the surfactant-free control (72 ± 5%, *p* > 0.05, Table 7). This demonstrates that the presence of neutral polyoxyethylene-based micelles neither hindered reagent diffusion nor compromised the accessibility of reactive amino groups on the protein surface, standing in sharp contrast to the near-complete electrostatic inhibition exerted by anionic SDS.

Beyond global conformational effects, surfactants can critically modulate bioconjugation kinetics by altering local microenvironmental polarity and introducing steric barriers to PEG accessibility. Anionic surfactants like SDS are well-known to cooperatively bind protein backbones into micelle-like clusters (“necklace-and-bead” model),^77,78^ which imposes severe steric shielding around surface lysines and alters the local dielectric environment and nucleophile solvation, ultimately suppressing productive encounters with activated PEG.^79^ In contrast, neutral polysorbate 20 does not form such high-affinity backbone complexes;^80^ its micellar assemblies leave the local hydration shell and steric accessibility of reactive amino groups largely intact. Consequently, the bimolecular collision frequency between mPEG-SC and protein nucleophiles is preserved, explaining why polysorbate 20 maintains a conjugation conversion statistically indistinguishable from the surfactant-free aqueous medium (Table 7).

## 4 Conclusion

This study elucidates the critical role of the physicochemical environment in modulating PEGylation efficiency. Among the additives evaluated, DMSO proved most effective for maximizing yield, a result driven by its high dispersion cohesion parameter, which optimizes PEG solvation and reduces competitive hydrolysis. Furthermore, while nonfunctionalized PEG of intermediate molecular weights can promote higher-order conjugation via the excluded volume effect, high-molecular-weight variants or anionic surfactants like SDS act as inhibitors due to viscosity and electrostatic repulsion, respectively.

From a process-development perspective, these findings highlight the reaction medium as an additional parameter that can be considered when optimizing PEGylation process. The effects observed for organic cosolvents, nonfunctionalized PEGs and surfactants demonstrate that these components can modulate PEGylation efficiency through distinct physicochemical mechanisms. Therefore, appropriate selection of reaction-medium components may provide an additional means of improving conjugation efficiency, controlling the distribution of PEGylated species and reducing unnecessary consumption of activated PEG. These considerations may be particularly relevant during process development and scale-up, where reaction-medium composition can affect protein stability, molecular interactions and overall process performance. Taken together, these findings enable the formulation of practical design rules for medium engineering in protein PEGylation:

(i) **Cosolvent selection**: The Relative Energy Difference (RED) from Hansen solubility parameters can serve as a predictive screening tool, where polar aprotic solvents with high dispersion cohesion (δ_d_ = 18.4 MPa^1/2^, such as DMSO) optimize polymer solvation and suppress competitive hydrolysis;
(ii) **Surfactant compatibility**: Nonionic polyoxyethylene surfactants (e.g., polysorbate 20) above their CMC should be favored for stabilization against aggregation without compromising reaction kinetics or steric accessibility, whereas ionic surfactants (e.g., SDS) must be avoided due to severe electrostatic inhibition; and
(iii) **Macromolecular crowding**: Nonreactive PEGs of intermediate molecular weight (4–8 kDa) can be exploited to modulate conjugate distribution via excluded volume effects, while high-molecular-weight variants (≥20 kDa) should be excluded to prevent diffusion and mass-transfer limitations.
